# Whole Genome Sequencing of *Klebsiella variicola* Strains Isolated from Patients with Cancer

**DOI:** 10.3390/antibiotics14080735

**Published:** 2025-07-22

**Authors:** Alicja Sękowska, Andrés Carrazco-Montalvo, Yulian Konechnyi

**Affiliations:** 1 Department of Microbiology, Ludwik Rydygier Collegium Medicum in Bydgoszcz, Nicolaus Copernicus University in Toruń, 85-094 Bydgoszcz, Poland; 2 Department of Clinical Microbiology, Dr. A. Jurasz University Hospital No. 1 in Bydgoszcz, 85-094 Bydgoszcz, Poland; 3 Centro de Referencia Nacional de Genómica, Secuenciación y Bioinformática, Instituto Nacional de Investigación en Salud Pública “Leopoldo Izquieta Pérez”, Quito 170403, Ecuador; acarrazco@inspi.gob.ec; 4 Department of Microbiology, Danylo Halytsky Lviv National Medical University, 79010 Lviv, Ukraine; konechnyi_yulian@meduniv.lviv.ua

**Keywords:** antimicrobial resistance genes, *Klebsiella variicola*, oncologic patients, virulence genes, whole genome sequencing

## Abstract

**Background:** *Klebsiella variicola* is a Gram-negative, capsulated, nonmotile, facultative anaerobic rod. It is one of the species belonging to the *K. pneumoniae complex*. The objective of this study was to gain insights into the antimicrobial resistance and virulence of *K. variicola* strains isolated from clinical samples from oncologic patients. **Methods:** Strain identification was performed using a mass spectrometry method. Whole genome sequencing was conducted for all analyzed strains. Antimicrobial susceptibility was determined using an automated method. The presence of antimicrobial resistance mechanisms and genes encoding extended-spectrum beta-lactamases (ESBL) was assessed using the double-disc synergy test and genotypic methods. **Results:** All isolates were identified as *K. variicola* using mass spectrometry and whole genome sequencing (WGS). All isolates were ESBL-positive, and two of them harbored the *bla*_CTX-M-15_ gene. In our study, the *bla*_LEN-17_ gene was detected in all strains. Genome sequence analysis of the *K. variicola* isolates revealed the presence of virulence factor genes, including *entAB*, *fepC*, *ompA*, *ykgK*, and *yagWXYZ*. Two different plasmids, IncFIB(K) and IncFII, were identified in all of the analyzed *K. variicola* strains. The detected virulence factors suggest the ability of the bacteria to survive in the environment and infect host cells. All isolates demonstrated in vitro susceptibility to carbapenems. **Conclusions:** Further studies are needed to confirm whether multidrug-resistant *K. variicola* strains represent an important pathogen in infections among oncologic patients.

## 1. Introduction

*Klebsiella variicola* is a Gram-negative bacteria that belongs to the *Enterobacteriaceae* family. Initially, *K. variicola* was often misclassified as *Klebsiella pneumoniae* due to its close resemblance, but advances in mass spectrometry and sequencing techniques have enabled differentiation between these species [1]. With appropriate identification methods, human infections caused by *K. variicola* are increasingly being recognized [2]. The name “*variicola*” is derived from the Latin va.ri.i’co.la., meaning “inhabitant of different places” [3]. These bacteria can be found in a variety of environmental sources worldwide, including water, soil, sewage, plants, and animals [3,4]. For many years, *K. variicola* was generally considered less virulent than *K. pneumoniae*. However, a recent study by Long et al. [1], using whole genome sequencing (WGS) revealed that *K. variicola* frequently causes severe infections similar to those caused by *K. pneumoniae*. In humans, *K. variicola* usually causes bacteremia, urinary tract infections, as well as respiratory tract infections [1,5,6,7,8,9,10,11,12]. The *Klebsiella* genus may cause infections in immunocompromised patients, including those with cancer. Some authors emphasize the important role of *K. pneumoniae*, while others highlight the emerging significance of *K. variicola* causing infections in cancer patients, particularly those with solid tumors [9,13,14,15]. Infections are a well-known cause of morbidity and mortality in cancer patients due to their compromised immune systems. They also represent one of the most common complications in this patient group.

During a 3-year study (2020–2022), *K. variicola* strains were isolated from 70 patients. Cancer was confirmed in 31 (44.3%) of them. Within this group, adults (>18 years) predominated, accounting for 24 (77.4%) cases. The most common diagnosis in patients with *K. variicola* infection was gastrointestinal cancer, found in 17 (54.8%). *K. variicola* is usually susceptible to antibiotics, but some authors have reported that increasingly multidrug-resistant *K. variicola* strains can cause infections in hospitalized patients and lead to outbreaks [16,17,18,19,20].

Therefore, it is necessary to monitor *K. variicola* infections in oncology patients. In this study, we performed a comparative genomic analysis of *K. variicola* strains isolated in Poland, identified using whole genome sequencing and mass spectrometry. Moreover, we detected antibiotic resistance and virulence genes in the *K. variicola* strains.

## 2. Results

Thirty one *K. variicola* strains were isolated from cancer patients, and three of them were selected for genomic analysis. The selection criteria included isolation in the same year (2022), multidrug resistance, and ESBL production.

*K. variicola* infections in adult cancer patients occurred over three times more frequently than in children. Among adults, infections were observed three times more often in men than in women. In the pediatric group, however, no gender-related differences in infection incidence were noted.

### 2.1. Patient’s Description

All three patients were hospitalized in 2022 at the same hospital, but in different departments. Detailed data regarding the cancer patients with *K. variicola* infection are presented in Table 1.

### 2.2. Identification and Susceptibility to Antimicrobials

For all three strains, a score of 2.2 was obtained using the mass spectrometry method. In the double-disk synergy test, all strains produced ESBL and were classified as multidrug resistant based on susceptibility results (Table 2).

### 2.3. Genome Sequencing

**Figure 1:**
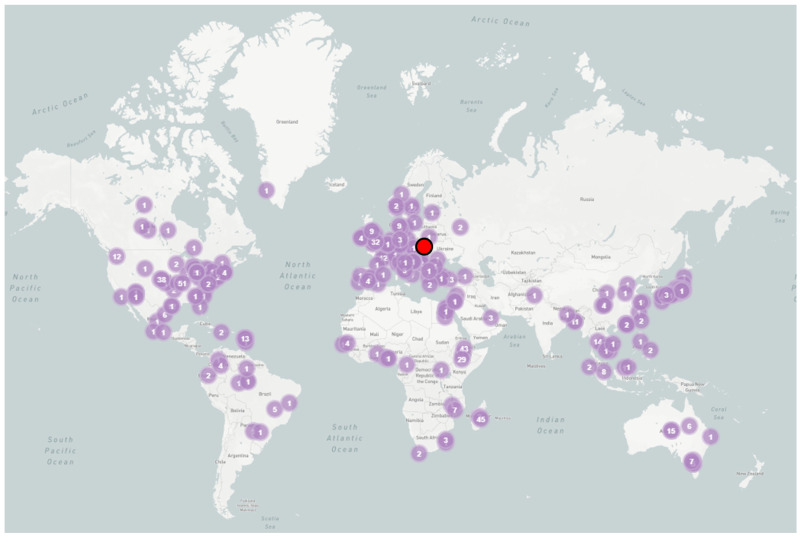
Worldwide distribution of *K. variicola*. The records from Poland are marked in red.

According to Pathogen.watch data, *K. variicola* is distributed across six continents. This study represents the first report of *K. variicola* in Poland confirmed by EZbiocloud (Figure 1).

ANI Using ANI and Kleborate v3, we found the following statistics per sample:

- MEDLv-3934_AS—Identity: 99.08%, # Contigs: 28, Genome size: 5,701,952 bps;
- MEDLv-3958_AS—Identity: 99.08%, # Contigs: 28, Genome size: 5,711,018 bps;
- MEDLv-3793_AS—Identity: 99.07%, # Contigs: 18, Genome size: 5,870,980 bps.

The GC content of the genome was 57.1% with a coverage of 100×.

Our samples were assigned to sequence type ST-1563, and the allelic profile was as follows: glyceraldehyde 3-phosphate dehydrogenase, translation initiation factor 2, malate dehydrogenase, phosphoglucose isomerase, phosphoporine E, beta-subunit of ribonucleic acid polymerase B, and periplasmic energy transducer, corresponding to the allelic numbers: 16-24-21-27-194-33-259.

Our sequence, along with 56 other *Klebsiella quasipneumoniae* sequences identified as ST-4891563, was included in the analysis. A core genome phylogenetic analysis was performed to identify closely related sequences using Roary v3.11.2 from the Pan Genome Pipeline (https://github.com/sanger-pathogens/Roary, accessed on 10 March 2023). For phylogenetic analysis, a single nucleotide polymorphism (SNP) approach was employed. The ABRicate bioinformatics pipeline was used to search for antimicrobial resistance genes via the CARD database (doi:10.1093/nar/gkw1004) and the Resfinder database (doi:10.1093/jac/dks261). Additionally, virulence factors were identified using the VFDB database within ABRicate. A threshold of 80% for both coverage and identity was applied to identify resistance and virulence genes. ABRicate and all associated databases were updated as of 14 January 2025.

**Figure 2:**
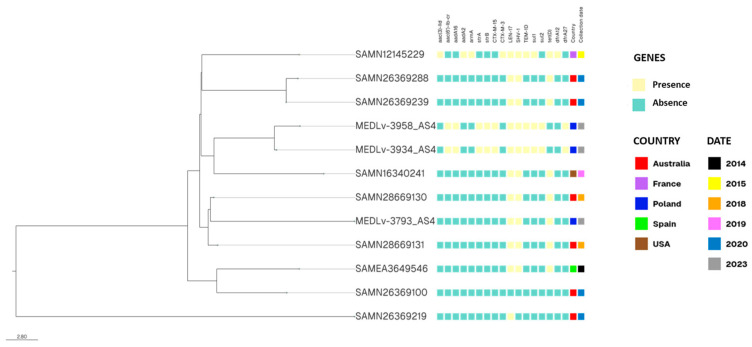
Phylogenetic analysis and metadata of samples assigned to sequence type ST-1563.

Figure 2 illustrates the phylogenetic tree and antimicrobial resistance profiles of *K. variicola* samples genetically closest to those reported in this study. Our isolates are highlighted in red. MEDLv-3958_AS and MEDLv-3934_AS carry resistance genes including aac(6′)-lb-cr, aadA16, strA, strB, CTX-M-15, LEN-17, TEM-1D, SHV-1, sul1, sul2, and dfrA27, forming a genetic cluster with sample SAMN16340241, collected in the USA in 2019. In contrast, our isolate MEDLv-3793_AS harbored resistance genes LEN-17, SHV-1, and tet(D), clustering with samples SAMN28669130 and SAMN28669131, collected in Australia in 2018.

All of the analyzed *K. variicola* strains carried *bla*_LEN-17_ (a chromosomal-based beta-lactamase gene) and *bla*_SHV-1_ (a broad-spectrum beta-lactamase gene). Additionally, all three strains possessed the *fosA* gene, which confers resistance to fosfomycin; however, only one strain exhibited in vitro resistance to fosfomycin. Furthermore, two of the *K. variicola* isolates (MEDLv-3934_AS and MEDLv-3958_AS) carried *bla*_CTX-M15_ (an extended-spectrum beta-lactamase gene), blaTEM-1D (a broad-spectrum beta-lactamase gene), *sul1* and *sul2* (mediating resistance to sulfonamides), as well as aadA16 and aac(6′)-Ib-cr genes (mediating resistance to aminoglycosides). Moreover, one strain (MEDLv-3793_AS) harbored the *tetD* gene, conferring resistance to tetracyclines. One of the analyzed strains (MEDLv-3958_AS) contained the *phoQ* gene, known to mediate resistance to colistin, but did not possess plasmid-borne colistin-resistance *mcr* genes.

PlasmidFinder identified two distinct plasmid replicon types among the analyzed strains: IncFIB(K) was detected in all strains, while IncFII was found in strains MEDLv-3958_AS and MEDLv-3934_AS.

Virulence genes were detected in all analyzed *K. variicola* strains, primarily associated with adhesion (Table 3).

These included the enterobactin genes *ent* and/or *fepC*, which are involved in iron acquisition; the *ompA* gene, associated with cell invasion; and the *ykgK* and *yag* genes, linked to adherence to epithelial cells and fimbriae formation. Additionally, virulome analysis revealed the presence of the pili gene *yag*, known for its role in adhesion.

## 3. Discussion

Since its first description in 2004 [21], only a few cases of clinical infections caused by *K. variicola* in cancer patients have been reported. Among these, Seki et al. [22] reported the first case of *K. variicola* infection presenting as sepsis in a 67-year-old woman with sinus cancer. Fontana et al. [23] identified a case of *K. variicola* and *Escherichia coli* bacteremia in a 72-year-old patient with colorectal cancer, initially misidentified as *K. pneumoniae*. Additionally, Dahl et al. [9] reported a case of *Clostridium perfringens* and *K. variicola* sepsis in a 68-year-old patient with pancreatic cancer. Conversely, Harada et al. [13], described 15 cases of *K. variicola* infections in patients with solid tumors, with colorectal cancer being the most frequent at nearly 27%, while tumors of the gastrointestinal tract accounted for over 73% of the cases. Similarly, our study observed a high prevalence of gastrointestinal tract tumors among cancer patients, accounting for 68% of cases. In contrast, Maatallah et al. [5], reported only 11.8% of *K. variicola* infections in patients with gastrointestinal cancer, with hematological malignancies being the most common at 23.5%. In the present study, nearly 20% of patients with *K. variicola* infections had leukemia. Moreover, Maatallah et al. [5] reported a very high mortality rate of 29.4%, which was higher than that observed in *K. pneumoniae* infections (13.5%).

In this study, we characterized three clinical *K. variicola* strains using WGS, highlighting the diverse types of infections caused by *K. variicola* in oncologic patients. The increasing availability of MALDI-TOF MS in microbiology laboratories is expected to improve and increase the frequency of *K. variicola* identification in the coming years. However, some authors suggest that sequencing remains the most reliable method for accurate identification of *K. variicola* strains. In our study, the *bla*_LEN_ gene was detected in all strains. This gene serves as a species-specific marker for *K. variicola* isolates, with all strains carrying the *bla*_LEN-17_ variant. Potter et al. [4] analyzed 145 *K. variicola* isolates and found that the most frequent variants were *bla*_LEN-16_, *bla*_LEN-24_, and *bla*_LEN-2_, detected in 84.2% of strains, while *bla*_LEN-17_ was present in only 5.5% of isolates. Conversely, Long et al. [1] analyzed 13 *K. variicola* strains and reported *bla*_LEN-24_ as the most common variant; none of the isolates harbored *bla*_LEN-17_.

All of the studied *K. variicola* strains were ESBL-positive in the double-disc synergy test; however, WGS revealed the presence of the ESBL gene *bla*_CTX-M15_ in only two strains. WGS-based analysis of antimicrobial resistance (AMR) genes also identified other resistance determinants in the isolates, specifically *bla*_SHV-1_ and *bla*_TEM-1D_, both detected in two strains. These genes encode broad-spectrum beta-lactamases located on plasmids. In the literature, several studies reported *K. variicola* strains producing ESBL enzymes such as SHV-12, SHV-187, CTX-M1, and TEM-1A; however, none of the isolates were obtained from cancer patients [10,24,25,26]. In the aforementioned studies, the authors described only single ESBL-positive *K. variicola* strains.

Two of the three analyzed strains exhibited phenotypic resistance to aminoglycosides, consistent with the presence of genes encoding aminoglycoside-modifying enzymes including aadA16ANT(3″), aac(6′) –Ib-cr5, strA, and strB. Similarly, phenotypic resistance to other antimicrobials such as trimetoprim, sulphonamides, and tetracyclines was supported by the detection of the corresponding resistance genes, including *sul1, sul2*, and *tetD*, respectively. The *fosA* gene, which confers resistance to fosfomycin, was identified in all three strains; however, phenotypic resistance was observed in only one strain. In turn, Wareth et al. [27] reported that 67% of *K. pneumoniae* strains carried the *fosA* gene; however, only one of them was phenotypically resistant to fosfomycin. Similarly, Wang et al. [28] described the presence of the *fosA7* gene in their isolates, which remained susceptible to fosfomycin, whereas strains carrying the *fosA3* gene were resistant to this antibiotic. The lack of correlation between the presence of a resistance gene and the phenotypic expression of resistance may be attributed to various factors, including gene silencing, regulatory mechanisms, or other genetic or environmental influences affecting gene expression [28]. In this study, we did not determine a variant of the *fosA* gene; therefore, further research is needed. Only one strain was resistant to colistin, and the *phoQ* gene was detected in this isolate.

Two different plasmid replicon types, IncFIB(K) and IncFII, were identified in all three analyzed *K. variicola* strains. These plasmids were found in combination but were not associated with antimicrobial resistance. In contrast, Saxenborn et al. [29] detected the IncFII plasmid in 40% of *K. variicola* isolates. These plasmid types were previously reported as predominant in *Klebsiella* species and are often found together [26,30,31]. Wareth et al. [27] reported that IncFIB (K) and IncFII plasmids were predominant in *K. pneumoniae* strains, detected in 67% and 54% of isolates, respectively. Furthermore, Morales-León et al. [25] suggested that the presence of the IncFII plasmid in hypervirulent *K. variicola* strains is possible.

Iron is recognized as an essential element for bacterial metabolism, survival, and pathogenicity. In our study, the genes *entAB* and *fepC*, encoding ferric enterobactin transport proteins, were detected in three and two strains, respectively. Similar results were obtained by Wareth et al. [27]. The authors analyzed 24 *K. pneumoniae* strains isolated from milk powder and found the *entB* and *fepC* genes in all strains. In turn, the *entA* gene was noted in 87.5% of the strains. However, Muraya et al. [26] observed the *entD* gene in only one *K. variicola* strain. Farzana et al. [16] suggested that the presence of the *entABCDEFHIJ* genes is associated with a hypervirulent phenotype. Furthermore, the *ompA* gene, noted in our strains, is considered an important cellular adhesion factor in *Klebsiella* rods, facilitating adhesion to epithelial and endothelial cells of both human and animal origin. Additionally, *ompA* has been implicated as a stress-response-encoding gene.

The *ykgK* gene plays a crucial role in binding to abiotic surfaces and serves as a major adhesive structure in biofilm formation [32]. In our study, all three strains were found to possess this gene, consistent with the findings of Folgori et al. [32], who detected it in all 11 analyzed *K. variicola* strains. On the other hand, Muraya et al. [26] observed adhesion genes in all 15 *K. variicola* isolates they analyzed, although these genes differed from those identified in our study.

An interesting topic is hypervirulence. In the available literature, a lot of information concerns *K. pneumoniae* strains, while data on *K. variicola* with this phenotype are limited. Farzana et al. [16] noted an association between the presence of the genes *kfuABC* and *entABCDEFHIJ* (both siderophores) and hypervirulent *K. variicola (KVAhv)* strains. Similarly, Morales-León et al. [25] detected the presence of *kfuABC*, *iutA*, *fimABC*, and *mrk ABCDF* in one *KVAhv* strain. In contrast to the above mentioned authors, Matsuda et al. [17], analyzed 421 *K. variicola* strains, but identified only 5 (1.2%) *KVAhv* isolates. In these strains, the following virulence genes were detected: *magA* (associated with the K1 type), *rmpA* (regulator of the mucous phenotype A), *iroB* (salmochelin), and *peg344* (metabolic transporter). In the present study, only the *entAB* genes were detected in the analyzed strains.

Our investigation highlights the value of WGS for precise species identification. However, several limitations of this study should be acknowledged. Firstly, this is a single-center study, the generalizability of our findings may be limited. Nevertheless, we analyzed genomes available in GenBank to provide the most comprehensive information possible. Secondly, the relatively small sample may have reduced the statistical power, particularly in the subgroup of oncologic patients. Despite these limitations, the study provides valuable insights into the clinical significance of *K. variicola*, and larger scale investigations are warranted to validate and expand upon these findings.

Our cases described various types of infections in cancer patients caused by *K. variicola*. In conclusion, we analyzed *K. variicola* strains collected from diverse clinical samples of cancer patients. The identified virulence factors indicated the bacteria’s adaptability to environmental persistence and host-cell infection. All isolates demonstrated in vitro susceptibility to carbapenems, novel beta-lactam/beta-lactamase inhibitor combinations, and amikacin. However, all strains were classified as multidrug resistant.

## 4. Materials and Methods

### 4.1. Bacterial Strains

Over the past three years, *K. variicola* strains were isolated from 70 patients. Among these, 25 (80.6%) were diagnosed with solid tumors, while 6 (19.4%) had leukemia. The majority of solid tumors (68.0%) were located in the gastrointestinal tract, with pancreatic tumors being the most prevalent (40.0%). The primary sources of *K. variicola* infection were blood and urine, together accounting for nearly 48% of cases. Detailed characteristics of cancer patients with *K. variicola* infection are presented in Table 4.

For detailed analysis, three *K. variicola* strains isolated from different clinical samples from three patients were selected: a 65-year-old woman, an 83-year-old woman, and a 3-year-old child—all within a six-month period in 2022. All patients had a history of cancer, specifically liver tumor, gallbladder tumor, and brain tumor, respectively.

### 4.2. Identification and Susceptibility to Antimicrobials

The strains were identified three times using MALDI-TOF MS (Bruker, Bremen, Germany) and confirmed by WGS. Antimicrobial susceptibility testing was performed using the Phoenix M50 (Becton-Dickinson, NY, USA) with NMIC-402 panels. Susceptibility breakpoints for ceftazidime-avibactam and meropenem-vaborbactam were determined using gradient diffusion strips (Liofilchem, Abruzzo, Italy). The minimum inhibitory concentration (MIC) of colistin was determined by microdilution method using the MIC COL test (Diagnostics Inc., Slovakia), while the MIC of fosfomycin was determined by the macrodilution method using AD Fosfomycin (Liofilchem, Italy). MIC values for all antimicrobials were interpreted according to EUCAST recommendations [33].

ESBL activity was detected using the disc diffusion method—specifically, the double disc synergy test with ceftazidim (30 µg), cefotaxime (30 µg), cefepime (30 µg) and amoxicillin-clavulanic acid (30 µg) (Liofilchem, Italy). *K. pneumoniae* ATCC 700603 (ESBL-positive) was used as the control strain.

### 4.3. Whole Genome Sequencing

For library preparation, 300 ng of genomic DNA was used following the DNA PCR-Free Prep protocol (Illumina, CA, USA), in accordance with the manufacturer’s guidelines. Paired-end sequencing (2 × 150 bp) was performed on the Illumina NovaSeq platform (Illumina, CA, USA). The resulting FastQ files underwent quality assessment using the FastQC tool. Adapter sequences, duplicates, and low-quality sequences reads were removed using Trimmomatic. Subsequently, de novo genome assembly was carried out using SPAdes v3.11.1, and the resulting contigs were mapped to the *K. variicola* reference strain F2R9 (ATCC BAA-830) [34]. For species identification and confirmation, we used the EZbiocloud platform (https://www.ezbiocloud.net/tools/ani, accessed on 28 April 2024) to perform average nucleotide identity analysis. Sequence statistics and multilocus sequence typing were determined using the Kleborate v3 platform (https://github.com/klebgenomics/kleborate, accessed on 1 April 2024). The resulting sequences were deposited in GenBank under BioProject Number PRJNA1071560.

To analyze the global distribution of the bacterium, the sequence was examined using the Pathogen.watch server (https://pathogen.watch/, accessed on 28 April 2024).

All *K. variicola* sequences collected between 2014 and 2023 from various countries and sequence types, as well as all sequences corresponding to ST 1563 (assigned to our isolates) were aggregated. Core genome phylogenetic analysis was performed using Roary, the Pan Genome Pipeline to identify sequences closely related to those reported in this study [35]. The ABRicate bioinformatics pipeline was used to identify antimicrobial resistance genes through the ResFinder database (doi:10.1093/jac/dks261) and virulence factors were identified using the VFDB database. Plasmid replicons were detected using PlasmidFinder (version 2.1), a tool integrated within the ABRicate platform. This tool was used to detect plasmid replicons based on sequence similarity. The analysis was conducted using default parameters, and the results were further validated by cross-referencing with the National Center for Biotechnology Information plasmid database.

## Figures and Tables

**Table 1:** The clinical characteristics of the patients.

Clinical Manifestation on Admission, Clinic, Age, Gender, Strain Number	First Source of Isolation	Other Source of Isolation	Antibiotics Used And Time	Clinical Sequalae
Peritonitis, Surgery, after 18 h, Intensive Care Unit, 65, woman, MEDLv-3958_AS	Wound swab, with *Enterococcus faecium*, *Candida albicans*	Blood, blood catheter, monocultures, peritoneal fluid with *Candida glabrata*	Imipenem, colistin, fluconazole 6 days	Death after 18 days
Gastrointestinal bleeding, Surgery, 83, woman, MEDLv-3934_AS	Blood catheter, monoculture	Drain into the hepatic duct, monoculture	Cefuroxime 3 days, next ceftriaxone 7 days, next imipenem 8 days, next vancomycin, colistin and fluconazole 12-12-10 days	Death after 22 days
Vomiting, nausea, abdominal pain, Pediatrics, hematology and oncology, 3, male, MEDLv-3973_AS	Urine, monoculture (>10^5^ CFU/mL)	-	Co-trimoxazole 4 days	Recovery

**Table 2:** Identification and susceptibility to antibiotics of *K. variicola* strains.

Antibiotic	MIC Value mg/L of *K. variicola* Strains (Interpretation)		
	MEDLv-3958_AS	MEDLv-3934_AS	MEDLv-3793_AS
Amoxicillin-clavulanic acid	>32/2 (R)	>32/2 (R)	>32/2 (R)
Piperacillin-tazobactam	>64/4 (R)	>64/4 (R)	>64/4 (R)
Ceftriaxone	>4 (R)	>4 (R)	>4 (R)
Ceftazidime	>8 (R)	>8 (R)	>8 (R)
Cefepime	>16 (R)	>16 (R)	>16 (R)
Imipenem	1 (S)	0.5 (S)	1 (S)
Meropenem	0.125 (S)	0.125 (S)	0.125 (S)
Gentamicin	4 (R)	>4 (R)	1 (S)
Amikacin	4 (S)	4 (S)	4 (S)
Tobramycin	>4 (R)	>4 (R)	2 (S)
Ciprofloxacin	0.25 (S)	0.25 (S)	0.5 (I)
Levofloxacin	0.5 (S)	0.5 (S)	1 (I)
Co-trimoxazole	>4/76 (R)	>4/76 (R)	1/19 (S)
Fosfomycin	129 (R)	32 (S)	16 (S)
Colistin	64 (R)	0.25 (S)	0.5 (S)
Ceftazidime-avibactam	0.5 (S)	0.5 (S)	0.25 (S)
Meropenem-vaborbactam	0.023 (S)	0.75 (S)	0.023 (S)

S—susceptible, I—susceptible, higher exposure, R—resistant.

**Table 3:** Virulence factors of *K. variicola* strains.

Virulence Factors	*K. variicola* Strains		
	MEDLv-3934_AS	MEDLv-3958_AS	MEDLv-3793_AS
Siderophores/enterobactin	*entAB* *fepC*	*entAB* *fepC*	*entAB*
Outer membrane protein	*ompA*	*ompA*	*ompA*
Adhesion/fimbriae	*ykgK*	*ykgK*	*ykgK*
Adhesion/pili	*yagWXYZ*	*yagWXYZ*	*yagVWXYZ*

**Table 4:** Characteristics of patients with *K. variicola* infection (*n* = 31).

Characteristics	
Age (median, min max), (years)	42.8 (2–87)
**Gender:**	**No. (%)**
Male	16 (51.6%)
Female	15 (48.4%)
**Diagnosis:**	
Pancreatic tumor	10 (32.3%)
Leukemia	6 (19.4%)
Biliary tract tumor	3
Bladder tumor	3
Liver tumor	2
Gallbladder tumor	2
Kidney tumor	2
Brain tumor	2
Laryngeal tumor	1
**Department:**	
Surgery	14 (45.1%)
Pediatric hematology and oncology	7 (22.6%)
Urology	4 (12.9%)
Transplantology	2
Intensive Care Unit	2
Neurosurgery	1
Laryngology	1
**Source of infection:**	
Blood	9 (29.0%)
Urine	6 (19.4%)
Peritoneal fluid/abdominal fluid	5 (16.1%)
Bile	4 (12.9%)
Wound swab	3
Prosthesis	2
Pus	1
Bronchoalveolar lavage	1

## Data Availability

The data presented in this study are available on a reasonable request from the corresponding author.
